# Flower-like Highly Open-Structured Binder-Free Zn-Co-Oxide Nanosheet for High-Performance Supercapacitor Electrodes

**DOI:** 10.3390/molecules27154850

**Published:** 2022-07-29

**Authors:** Qasim Abbas, Sajid Hussain Siyal, Abdul Mateen, Majed A. Bajaber, Awais Ahmad, Muhammad Sufyan Javed, Patrick Martin, Nicolas Joly, Patrizia Bocchetta

**Affiliations:** 1 Department of Intelligent Manufacturing, Yibin University, Yibin 644000, China; kuyasirabbas@gmail.com; 2 Metallurgy and Materials Engineering Department, Dawood University of Engineering and Technology, Karachi 74800, Pakistan; sajid.hussain@duet.edu.pk; 3 Department of Physics and Beijing Key Laboratory of Energy Conversion and Storage Materials, Beijing Normal University, Beijing 100084, China; abdulmateen@mail.bnu.edu.cn; 4 Department of Chemistry, Faculty of Science, King Khalid University, Abha 61413, Saudi Arabia; mb@kku.edu.sa; 5 Departamento de Quimica Organica, Universidad de Cordoba, E14014 Cordoba, Spain; awaisahmed@gcuf.edu.pk; 6 School of Physical Science and Technology, Lanzhou University, Lanzhou 730000, China; 7 Unité Transformations & Agroressources, Univ Artois—UniLaSalle, F-62408 Béthune, France; patrick.martin@univ-artois.fr (P.M.); nicolas.joly@univ-artois.fr (N.J.); 8 Dipartimento di Ingegneria dell’Innovazione, Università del Salento, 73100 Lecce, Italy

**Keywords:** ZnCo_2_O_4_, micro-flowers, nickel foam, binder-free electrode, energy storage, bi-metallic oxides

## Abstract

Scientific research is being compelled to develop highly efficient and cost-effective energy-storing devices such as supercapacitors (SCs). The practical use of SC devices is hindered by their low energy density and poor rate capability due to the binding agents in fabricating electrodes. Herein, we proposed flower-like highly open-structured binder-free ZnCo_2_O_4_ micro-flowers composed of nanosheets supported in nickel foam (ZnCoO@NF) with improved rate capability up to 91.8% when current varied from 2 to 20 A·g^−1^. The ZnCoO@NF electrode exhibited a superior specific capacitance of 1132 F·g^−1^ at 2 A·g^−1^ and revealed 99% cycling stability after 7000 cycles at a high current density of 20 A·g^−1^. The improved performance of the ZnCoO@NF electrode is attributed to the highly stable structure of the micro/nano-multiscale architecture, which provides both the high conduction of electrons and fast ionic transportation paths simultaneously.

## 1. Introduction

Clean and renewable energy sources are in high demand due to current energy shortages and environmental concerns [1,2]. Supercapacitors (SCs) with a high power density and long cycle life with minimum pollution have attracted considerable interest [3] because of their intriguing advantages, for example, lightweight and outstanding power, which are in high demand in manufacturing smartphones and handy gadgets [4]. SCs usually store electrical charge on the electrode material’s surface by adsorption/desorption, known as electrochemical double-layer capacitors (EDLCs). In the second type, the near-surface area via reduction/oxidation states changes, and a reversible redox reaction occurs; this charge-storing mechanism is known as a pseudocapacitive mechanism [5]. Pseudocapacitors (PCs) have a higher capacitance and energy density than EDLC-based SCs, but it is necessary to upgrade PCs before they can be used in real-world applications [6]. The redox reactions governed by the electrode material’s multiple oxidation states significantly impact the performance of PCs [7]. Designing nanostructured electrode materials for PC with high redox-active sites and improved performance is challenging. In this context, interchanging more than one electron for every redox reaction is considered to be advantageous. The performance of PCs can be improved by preparing the multiple valance state materials with abundant electroactive sites [8]. Transition metal oxides have sparked interest in PCs over the last decade due to easy fabrication and cost-effectiveness [4]. Binary metal oxides possess more valance states and higher electrical conductivity than single metal oxides, up to two times higher, and exhibit better performance [9]. The poor performance of transition metal oxides may be due to their inability to conduct electricity. Following prior research, increasing the electrical conductivity of host oxides by doping them with metals or semi-conductor elements is essential for achieving high power density in SCs [10]. Thus, Zn doping significantly increases the electrical conductivity of ZnCo_2_O_4_, especially in comparison with Co_3_O_4_, and redox-inert Zn^2+^ offers good synergistic impacts and considerably improves the rate-performance of cobalt-oxide.

Cobalt-based binary metal-oxide electrode materials such as ZnCo_2_O_4_, FeCo_2_O_4_, CuCo_2_O_4_, NiCo_2_O_4_, and MnCo_2_O_4_ can significantly increase the performance of PCs [11,12,13]. It is believed that ZnCo_2_O_4_ is the most promising candidate due to the low toxicity of zinc metal and reasonably significant capacitance of Co_3_O_4_ (3560 F·g^−1^), and it also possesses good stability at low cost [14]. However, the rate performance of cobalt-based binary metal-oxides is poor, and for SCs, the rate–performance parameter is critical. To enhance the ZnCo_2_O_4_ electrode’s overall performance, three general strategies are typically employed: (i) development of nanostructured materials with a visually appealing structure and a large surface area; (ii) research and development of new composites with high conductivity, which includes carbon nanotubes and graphene; and (iii) enhancement of the SC device’s potential window, as energy density is inversely proportional to the potential window’s square (E = 1/2CV^2^) [15]. Increasing the capacitance and voltage of SCs can improve their energy density. Capacitance can be enhanced by increasing the electrode material’s surface area and pore size [16]. Binders prevent active materials from falling off during electrode operation by cohering the electrode material with substrates. When making an electrode, the binder should be able to provide the necessary strength and pore sizes. However, binding or cohesive agents inevitably cover some of the active material’s surface area or pores. As a result, the electrochemical performance of SCs is directly influenced by the binders and their content in the electrodes [17]. Binder-free electrodes have recently been found to significantly increase electrical conductivity and ionic diffusion paths and thus improve overall performance. In addition, the “dead volume” in the electrode material can be avoided using a binder-free electrode with a highly porous and robust construction [18].

Saravanakumar et al. fabricated a binder-free hierarchical electrode of ZnCo_2_O_4_, showing a suitable specific capacitance of 92 mF·cm^−2^ at 0.5 mA·cm^−2^ [19]. Fu et al. made a binder-free flower-like ZnCo_2_O_4_ electrode with a good capacitance of 689.4 F·g^−1^ at 1 A·g^−1^ with excellent cyclic stability of 97.1% after 1500 cycles [20]. Kamble et al. used ZnCo_2_O_4_ to make binder-free electrodes and obtained a good capacitance of 127.8 F·g^−1^ at 1 mA·cm^−2^ with excellent cycling stability of 3000 cycles [21]. Although considerable efforts to increase the capabilities and cycling stability of ZnCo_2_O_4_ have been ongoing, the cycling stability and rate performance are still crucial and need to be significantly improved. The electrochemical performance of ZnCo_2_O_4_ electrodes can be further improved by exploring the various morphologies. We propose that electrodes of ZnCo_2_O_4_ with micro/nano morphology can increase surface area and pore size, resulting in increased energy density and rate performance.

Herein, we proposed binder-free ZnCo_2_O_4_ micro-flowers composed of nanosheets supported on (ZnCoO@NF), improving the rate capability up to 91.8% when current varied from 2 to 20 A·g^−1^. The ZnCoO@NF electrode exhibits a superior capacitance of 1132 F·g^−1^ at 2 A·g^−1^ with excellent cycling stability of ~99% after 7000 cycles at a high current density of 20 A·g^−1^. The improved performance of the ZnCoO@NF electrode is attributed to the highly stable structure of micro/nano-architecture, which provides both high electronic conduction and fast ionic transportation paths.

## 2. Materials and Methods

### 2.1. Zn-Co-Oxide Synthesis on Nickel Foam

The 2D ZnCo-oxide micro-flowers assembled by nanosheets on nickel foam (NF) were grown directly using a low-cost and straightforward hydrothermal synthesis method. In a usual cycle, a homogenous solution was made by mixing 2 mmol of Zn(NO_3_)_2_·6H_2_O, 4 mmol of Co(NO_3_)_2_·6H_2_O, 2 mmol of NH_4_F, and 6 mmol urea in 40 mL of deionized water and ultra-sonicating the solution for 60 min. Afterwards, a hygienic piece of nickel foam (NF) was hung on the inside walls of a Teflon autoclave, and the solution mixture was injected into the Teflon autoclave. After securing the autoclave, it was placed in an electric oven and heated to 135 °C for 16 h. The NF was assembled with ZnCoO-oxide precursors, washed with deionized water and ethanol, and sonicated for 5 min to remove the remaining particles from the surface of NF. For further processing, the ZnCoO@NF was dehydrated in an air furnace for 12 h. To improve the crystallinity, the ZnCoO@NF precursor was heated for 2 h at 250 °C in the air atmosphere. The experiment was repeated twice and yielded the same results each time.

### 2.2. Fabrication of Electrode

The as-synthesized ZnCoO@NF can be directly used as an electrode for SCs without any binder. The ZnCoO@NF is cut into 1 × 1 cm^2^ pieces and directly used as a working electrode. Based on the difference in the weights of the bare NF and ZnCoO@NF, 1.26 mg·cm^−2^ of active material was found on the surface of NF.

### 2.3. Characterization of Material

Field emission scanning electron microscopy (SEM, FEI Nova 400, FEI Company, Hillsboro, OR, USA) and transmission electron microscopy (TEM, JEM 2100F TEM, Jeol, Tokyo, Japan) were both used to examine the materials’ microstructures and morphology. X-ray powder diffraction (XRD) (PANalyticalX’Pert Powder, Malvern Panalytical, Malvern, UK) was used to determine the crystal structure of the synthesized product. The X-ray source was an Escalab (250Xi) (Thermo Fisher Scientific, Waltham, MA, USA) with an Al Ka (1486.5 eV). The specific surface area calculation was made with a Quantachrome Instrument (Version 5.12, Boynton Beach, FL, USA) and the Brunauer-Emmett-Teller (BET) method.

### 2.4. Electrochemical Tests Measurements

The electrochemical tests, including cyclic voltammetry (CV), galvanostatic charge/discharge (GCD), and electrochemical impedance spectroscopy (EIS), were performed in an open-circuit position in a frequency ranging from 0.001 Hz to 100 kHz. All these tests were performed using a platinum sheet (counter electrode), Ag/AgCl (reference electrode), and ZnCoO@NF (working electrode) in a 6 KOH electrolyte at room temperature using an electrochemical workstation (CHI 660E, CH Instruments, Wuhan, China) in a three-electrode configuration. The specific capacitance (Cs) was calculated using the following Equation (1):

(1) $$\mathrm{Cs}=\frac{I\times \Delta t_{d}}{m\times \Delta V}$$

where C_s_ (F·g^−1^) represents the specific capacitances, I represents the current (A), Δt_d_ (s) represents the discharging time, m (g) represents the active mass of the ZnCoO material on NF, and the potential window is represented by ΔV (V).

## 3. Results

**Figure 1:**
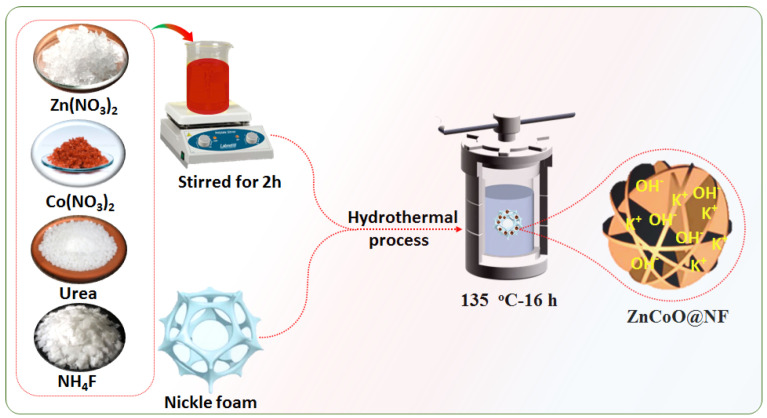
The schematic representation of the synthesis process of ZnCoO@NF through the hydrothermal method.

Figure 1 gives a schematic route for the fabrication process of ZnCoO on nickel foam. In order to improve the mechanical and crystalline, the ZnCoO was post-annealed for the short term at 250 °C for 2 h in an air atmosphere with a ramp rate of 2 °C·min^−1^.

**Figure 2:**
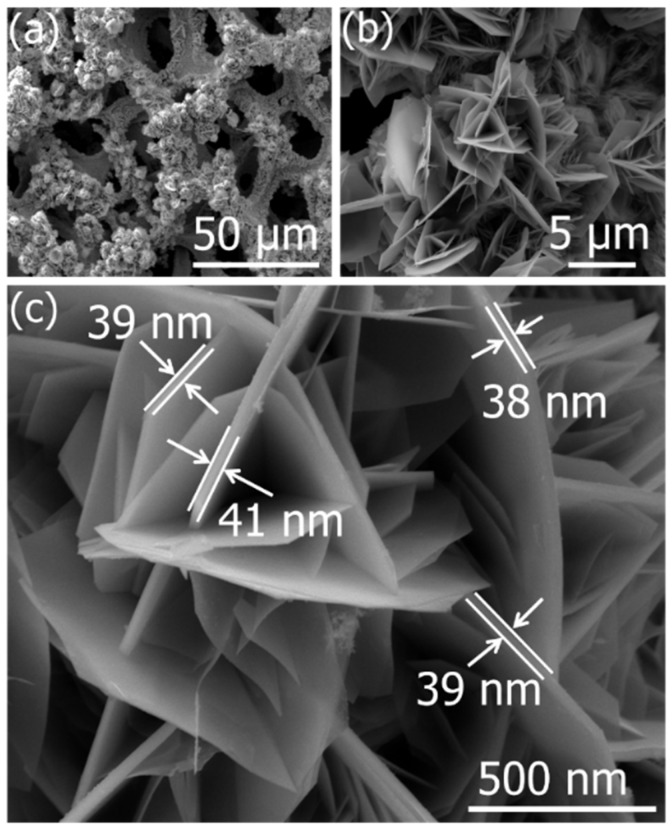
The SEM images of ZnCoO@NF at different magnifications: (**a**) low, (**b**) medium, and (**c**) high.

Figure 2a–c shows the morphology of as-prepared ZnCoO@NF at different magnifications, reflecting the 2D micro-flowers made by nanosheets. Figure 2a shows a low-resolution SEM image, demonstrating the rough surface with the growth of high-density ZnCoO nanosheet architecture on the entire surface of the NF and reflecting a compact design and interconnected structure. The high-magnification SEM images show that the various nanosheets make a micro-flower-like structure (Figure 2b). An open channel structure formed by interconnected nanosheets and an average thickness of 38 nm is shown in Figure 2c. Compared and a pure nanosheet, these micro-flower patterns increase the surface area of the electrode and enhance the electrode/electrolyte interface, making it better suited for use in SCs.

**Figure 3:**
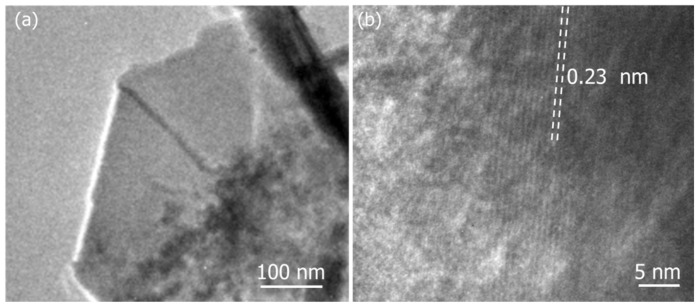
Typical (**a**) low-resolution and (**b**) high-resolution TEM images of the ZnCoO@NF nanosheets.

TEM analysis was further conducted to investigate the ZnCoO structure and surface morphologies in more depth. A characteristic TEM image with low resolution represents nanosheets of ZnCoO (Figure 3a,b). Figure 3a shows that a 2D structure can be derived from the interconnected nanosheets, increasing the intrinsic electronic conductivity. Figure 3b shows that the lattice fringe spacing is 0.23 nm, corresponding to the 111 planes of ZnCo_2_O_4_ (PDF# 23-1390). These findings are also in line with the results of the SEM. It should be noted that in the SEM and TEM images, we can see some residual nanoparticles. It is confirmed that there is no effect from these residual nanoparticles on the electrochemical performance of the electrode.

**Figure 4:**
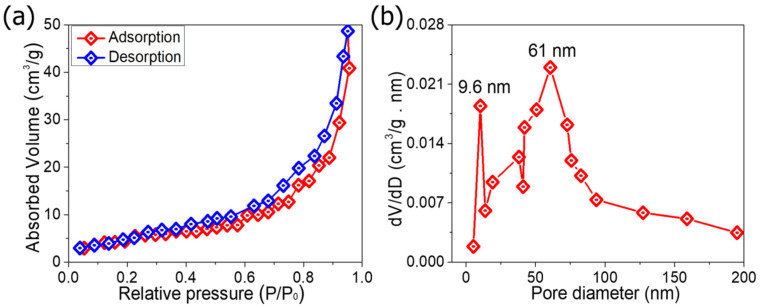
(**a**) N_2_ adsorption and desorption isotherms; (**b**) pore-size distribution of ZnCoO@NF.

Figure 4a shows the specific surface area and pore size distribution (PSD) of the ZnCoO@NF electrode recorded at 77.5 K. BET absorbed volume of ZnCoO@NF is about 50 cm^3^/g at 1.0 P/P_0_, which means that ZnCoO@NF has a highly porous structure (55 m^2^·g^−1^). Moreover, this study demonstrated an H1-type hysteresis loop, which suggested the presence of a mesoporous structure in the solution. The PSD was calculated from the desorption isotherm using the Barrett-Joyner-Halenda (BJH) method, as shown in Figure 4b. The PSD of ZnCoO@NF is centred at about 9.6 and 61 nm, reinforcing the prepared sample’s mesoporous structure with pore sizes ranging from 9.6 to 61 nm. These findings show that the ZnCoO@NF sample has a greater surface area that leads to efficient ion and electron shipping, implying that high electrochemical activity in the ZnCoO@NF electrode is expected. For efficient PC electrodes, a high specific surface area can effectively enhance the electrochemical performance and provide a large number of electroactive sites for PCs.

**Figure 5:**
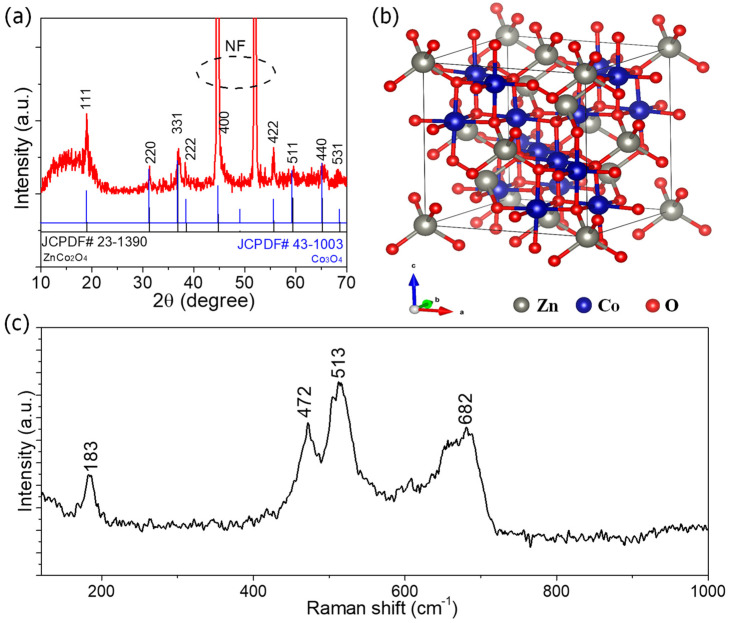
Physical characterizations of ZnCoO@NF: (**a**) xrd pattern, (**b**) crystal structure (**c**) Raman spectra.

The XRD pattern of ZnCoO@NF (Figure 5a) is well-indexed according to the standard spinel phase ZnCo_2_O_4_ (JCPDF# 23-1390). The particular diffraction peaks of ZnC_2_O_4_ located at 21°, 32°, 37°, 39°, 44.7°, 56°, 59.5°, and 66° correspond, respectively, to 111, 220, 311, 222, 422, 511, and 440 lattice planes. Compared with the standard ZnCo_2_O_4_ spinel, these results are consistent with the previous findings [20]. The lattice plane of 400 at 44.7° is overlapped with the XRD peak of NF. No other impurities are detected except two intense peaks from the NF at 45° and 52.5° of 2θ, demonstrating the high purity of the sample. The JCPDF# 23-1390 of pure Co_3_O_4_ was added as a comparison to confirm the difference between ZnCo_2_O_4_ and Co_3_O_4_. The diffraction peaks of as-prepared ZnCoO@NF clearly matched with JCPDF# 23-1390 and confirm the pure phase of ZnCo_2_O_4_. The crystal structure ZnCo_2_O_4_ nanostructures are shown in Figure 5b. Binary spinel metal oxide ZnCo2O_4_ with Co^2+^ at the tetrahedral sites (8a) in the spinel Co_3_O_4_ replaced by Zn^2+^ offers a better stability. In addition, the spinal structure can help increase the redox reaction sites, which boosts the specific capacitance of bimetallic oxide-based electrodes. Thus, choosing the bimetallic oxide and crystal structure is critical for overall performance and structural stability during the electrochemical discharge process [22].

The Raman spectrum further characterized the as-synthesized ZnCoO@NF to confirm the Raman band characteristics. From Figure 5c, the Raman spectrum of ZnCoO@NF demonstrates four active vibrational bands located at 183, 472, 513, and 682 cm^−1^ assigned to the characteristic F_2g_, E_g_, F_2g_, and F_2g_ phonon modes, respectively. The presence of Zn, Co, and O in these distinct peaks of the Raman spectrograms suggests the formation of ZnCo_2_O_4_. This indicates that the prepared sample is free of contaminants [23].

**Figure 6:**
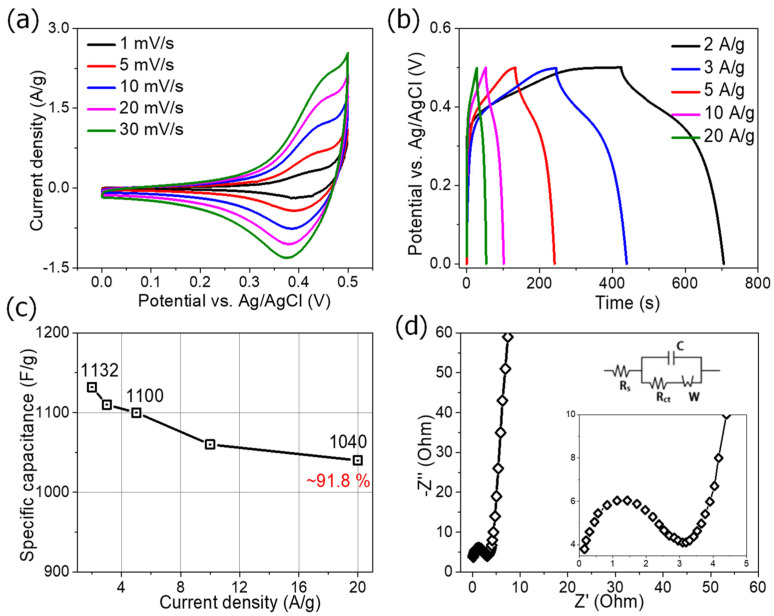
(**a**) CV curve at varied scan rates, (**b**) charge-discharge curve at current densities, (**c**) specific capacitance, and (**d**) electrochemical impedance spectra of the ZnCoO@NF electrode.

The electrochemical performance results of ZnCoO@NF are shown in Figure 6. The CV was performed at various scan rates in a potential window ranging from 0.0 to 0.5 V. The CV curves indicate two redox peaks at approximately 0.38 and 0.42 V (vs Ag/AgCl), which represent the typical pseudocapacitive behavior of the ZnCoO@NF associated with Co^3+^/Co^2+^ during the charge/discharge process [19]. The reduction peak shift is related to the internal resistance of the electrode materials, which further confirms the pseudocapacitive features with enhanced charge storage [24]. Moreover, the CV possesses a large area under the curve with high current response, indicating better charge storage behavior. The electrodes’ excellent redox reversibility can be seen in the redox peaks caused by reactions involving Co–O/Co–O–OH [25].

The electrochemical property of the ZnCoO@NF electrode was tested based on charging and discharging at various current densities to accurately estimate the charge storage capability. The GCD measurements of the ZnCoO@NF electrode were carried out in a potential window ranging from 0.0 to 0.45 V. Figure 6 depicts typical GCD curves of ZnCoO@NF at various current densities (2 to 20 A·g^−1^) (b). The GCD curves are nearly symmetric (Coulombic efficiency 98%), have apparent redox behavior, and agree well with the CV results, demonstrating the pseudocapacitive charge storage features. The charge and discharge curves primarily have voltage terraces at about 0.3 V and 0.36 V, respectively. Equation (1) can be used to calculate the specific capacitance Cs based on the curves. Based on Equation (1), the obtained specific capacitances of the ZnCoO@NF electrode are about 1132, 1120, 1100, 1060, and 1040 F·g^−1^ at current densities of 2, 3, 5, 10, and 20 A·g^−1^, respectively, as shown in Figure 6c. When the discharge current density changes from 2 to 20 A·g^−1^, the capacitance remains at 91.8%. The capacitance of the ZnCoO@NF electrode is much better than other electrodes such as Gd-doped CeOx nanoflowers (280 F·g^−1^ in 1 M NaOH at 1 V·s^−1^) [26] and mesoporous Co_3_O_4_ nanosheets on carbon foam (106 F·g^−1^ in 1 M NaOH) at a scan rate of 0.1 V·s^−1^ [27].

Because of its multi-channel porous nature and enhanced ionic transportation, the ZnCoO@NF demonstrated exceptional specific capacitance, far exceeding that of previously reported ZnCo_2_O_4_-based electrode materials.

The total capacitance of ZnCoO@NF can be attributed to the following significant contributions: (1) the micro-flowers composed of nanosheets with highly open structures supply abundant active sites for the Faraday reaction and offers easy diffusion paths for fast ionic transportation; (2) the electrochemical performance can be promoted by improving the specific area, pore size, and multi-channel nano/micro-architecture.

Furthermore, electrochemical impedance spectroscopy (EIS) was conducted to study the kinetics of charge transfer in the ZnCoO@NF electrode to confirm the charge transport properties. Figure 6d displays the Nyquist plots of the EIS spectra for the ZnCoO@NF electrode. The diameter of the semi-circle is used to estimate the charge transfer resistance (R_ct_ = 1.52 Ω), which means that ion diffusion and electrolyte penetration in the active materials are reduced. The solution resistance (R_s_ = 0.13 Ω) is the combination of the electrolyte’s and electrode’s internal resistance. The values of R_ct_ and R_s_ are smaller than in pristine Co_3_O_4_ (R_s_ = 1.32 Ω) [28] and ZnO (R_s_ = 2.82 Ω and (R_ct_ = 2.25 Ω) [29]. The high-sloped curve at the high-frequency region indicates the fast ionic diffusion with enhanced electroactive sites in the ZnCoO@NF electrode material. The Nyquist plot was fitted with an equivalent circuit using ZSim view software, as presented in the inset of Figure 6d. The equivalent circuit shows R_s_, R_ct_, C (the double layer capacitance), and W (the Warburg diffusion element). Hence, the ZnCoO@NF electrode possesses more capacitive-type charge-storage features [30].

**Figure 7:**
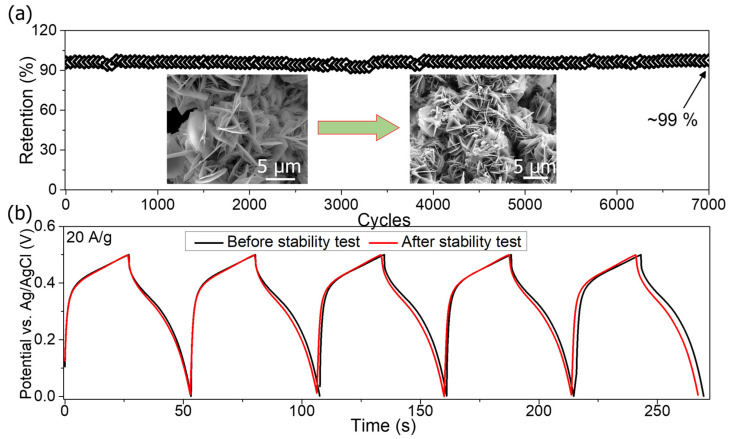
**(a**) Cycling stability tests of ZnCoO@NF electrode (**b**) Five GCD cycles before and after stability test.

Another vital parameter to consider when evaluating the practical applications of SCs is their cycling stability. Thus, the cycling stability test of the ZnCoO@NF electrode was performed by deliberately running it for 7000 charge/discharge cycles at a high current density of 20 A·g^−1^, and the results are shown in Figure 7a. After 7000 cycles, the specific capacitance remains at 99%. In the long-term cycling test, the ZnCoO@NF exhibited excellent charge stability and high performance, as shown by the obtained results. Moreover, at 20 A·g^−1^, the first and last five GCD cycles are shown in Figure 7b, indicating that the ZnCoO@NF electrode exhibits excellent stability before and after testing. Due to the large exposed surface area of its unique micro-flower composed of nanosheet architectures, the ZnCoO@NF electrode has impressive electrochemical performance. Micro-flowers can be grown directly onto the substrate without a binder, enabling direct electron intercalation between the substrate and the active material. It is easier to conduct a fast ion-diffusion reaction with the active material due to the micropores in the NF substrate. Because of their high rate capability, excellent capacitance, and admirable cycling stability, ZnCoO@NF electrodes are potential candidates for pseudocapacitive SC applications. The detailed comparisons of the current work with the previous literature in terms of electrode material, capacitance, current density, number of cycles, and retention are presented in Table 1.

## 4. Conclusions

The ZnCoO@NF was successfully synthesized using a straightforward hydrothermal method followed by a thermal procedure. The ZnCoO@NF electrode exhibits excellent electrochemical properties in an aqueous KOH electrolyte. The ZnCoO@NF electrode demonstrates a high specific capacitance of 1132 F·g^−1^ at 2 A·g^−1^ and 1040 F·g^−1^ at 20 A·g^−1^ with improved rate capability up to 91.8% when the current increased from 2 to 20 A·g^−1^. The ZnCoO@NF electrode shows excellent cycling stability of ~99% after 7000 cycles at a high current density of 20 A·g^−1^. Future energy storage devices will benefit from the new binder-less electrode material with high specific capacitance and rate capability. In light of the promising results, SCs combined with other energy conversion/storage devices may be commercially available soon.

## Figures and Tables

**Table 1:** Comparisons of the ZnCoO@NF electrode with previous literature in terms of electrode material, capacitance, current density, number of cycles, and retention.

Serial No.	Electrode/Materials	Capacitance (F·g^−1^)	Current Density (A·g^−1^)	No. of Cycles (*n*)	Retention (%)	References
1	ZnCoO@NF	1132	2	7000	99	This work
2	ZnCo_2_O_4_	865.35	1.0	1000	73	[31]
3	ZnCo_2_O_4_/C composite	90.1	8.0	1000	96	[32]
4	ZnCo_2_O_4_ NWs/rGO	1116.6	2	5000	93.4	[33]
5	ZnCo_2_O_4_-microsphere	689	1.0	5000	98	[34]
6	ZnCo_2_O_4_ nanowires	694	2.0	2000	85	[35]
7	ZnCo_2_O_4_ nanoflowers	770	10	3000	89	[36]
8	ZnCo_2_O_4_ microspheres	647	1.0	3000	96	[23]
9	Flower-like ZnCo_2_O_4_	689	1.0	1500	97	[37]
10	ZnCo_2_O_4_ nanoparticles	710	1.0	3000	84	[38]

## Data Availability

The data presented in this study are available on request from the first author Qasim Abbas, who is responsible for the performed experiments.
